# 

**Published:** 1832-01-01

**Authors:** 


### MEDICO-CHIRURGICAL

### REVIEW,

AND

### JOURNAL

OF

### PRACTICAL MEDICINE.

*(NEW SERIES.)*

VOLUME SIXTEEN,

[1st of OCTOBER, 1831, to 31st of MARCH,]

1832.

VOL. XX. of ANALYTICAL SERIES.

not to be indexed

EDITED BY JAMES JOHNSON, M.D.

PHYSICIAN EXTRAORDINARY TO THE KING,

&c. &c. &c.

Surgeon General's Office LIBRARY 57455 Washington D.C.

brown

LONDON:

PUBLISHED BY S. HIGHLEY, 32, FLEET STREET,

AND WEBB STREET, ST. THOMAS'S HOSPITAL,

And all other Booksellers.

W1
ME75T

### BIBLIOGRAPHICAL RECORD;

OR,

*Works received for Review since last Quarter.*

---

1. Brand's Lunacy Case. A full Report of this most interesting and extraordinary Investigation, including copious Animadversions on the principal Actors in the Drama, &c. By CHARLES DUNNE, M.R.C.S. &c. Octavo, pp. 132. London, 1831.

---

2. The Principles of Lithotrity; or a Treatise on the Art of extracting the Stone without Incision. By Baron HEURTELOUP, Doctor of the Faculty of Medicine, Paris. Octavo, pp. 486, with numerous Plates. Whittaker, London, October, 1831.

---

3. Lectures on Population, Marriage, and Divorce, as Questions of State Medicine, &c. By MICHAEL RYAN, M.D. Small 8vo. pp. 72. Renshaw and Rush, 1831.

---

4. Elements of Surgery. By ROBERT LISTON, Senior Surgeon to the Royal Dispensary of Edinburgh, &c. Octavo, pp. 334. Part the Second. Edinburgh and London, 1831.

---

5. Cases of Insanity, with Medical, Moral, and Philosophical Observations and Essays upon them. By M. ALLEN, M.D. Part I. Vol. I. Octavo, pp. 212. 1831.

---

6. A Practical Guide to Operations on the Teeth; to which is prefixed, a Historical Sketch of the Rise and Progress of Dental Surgery. By JAMES SNELL, Dentist, M.R.C.S. Lecturer on Diseases of the Teeth, &c. Octavo, pp. 207. With Plates, 1831.

---

7. An Exposure of the continued Misrepresentations by Richard Phillips, Esq. in his Attempt to vindicate himself from Dr. Reid's first Exposure of his Misrepresentations, &c. By DAVID BOSWELL REID, M.D. F.R.S.E. Lecturer on Chemistry, &c. Octavo, pp. 16. Edinburgh, 1831.

☞ *Bella, horrida bella!*

---

8. Colour, Images in the Brain; with a View of the Bearings of their Detection on Philosophy: to which are added, Strictures on the Abstract of the Subject, printed by the Royal Soc. By J. FEARN, Esq. Octavo, pp. 39, sewed. Sept. 1831.

---

9. Observations on Cholera, as it appeared at Port Glasgow during the Months of July and August, 1831; illustrated by numerous Cases. By JOHN MARSHALL, M.D. Octavo, sewed, pp. 80. Oct. 1831.

---

10. General Remarks on the Health of English Manufacturers, and on the Need which exists for the Establishment of Convalescents' Retreats, as subservient to the Medical Charities of our large Towns. By JOHN ROBERTON, one of the Surgeons to the Manchester Lying-in Hospital, &c. Octavo, pp. 36. London, Oct. 1831.

---

11. A Manual of Midwifery, or Compendium of Gynæcology and Paidonosology; comprising a new Nomenclature of Obstetric Medicine, with a concise Account of the Symptoms and Treatment of the most important Diseases of Women and Children, and the Management of the various Forms of Parturition. Illustrated by Plates. By MICHAEL RYAN, M.D. &c. Third Edition, small 8vo. pp. 737. Renshaw and Rush, 1831.

☞ *This third edition is greatly enlarged, and now forms an extremely useful and cheap compendium of obstetrical knowledge.*

---

12. Operative Surgery. Maingault's Illustrations of the different Amputations performed on the Human Body; represented by Plates designed after Nature, with Alterations and practical Observations. By WILLIAM SANDS COX, Lecturer on Anatomy and Surgery at the Birmingham School of Medicine, and Surgeon to the General Dispensary. Folio, eight Plates, with Letter-press Explanations, &c.

---

13. A Synopsis of the Bones, Ligaments, Muscles, Blood-vessels, and Nerves of the Human Body. By WM. SANDS COX, Surgeon to the Dispensary, and Lecturer on Anatomy, &c. at the School of Medicine in Birmingham. Octavo, pp. 310, with five Plates. October, 1831, price 9s.

---

14. Essays on the Subjects of Iodine in Scrofulous Diseases; including an Inquiry into the Mode of preparing Iodaretted Baths. Translated from the French of M. LUGOL (Physician to the Hôpital St. Louis), by W. B. O'SHAUGHNESSEY, M.D. with an Appendix by the Translator, containing a Summary of Cases treated with Iodine, either simple or combined with Opium, Mercury, Lead, &c. Octavo, pp. 218. Highley, 32, Fleet Street, Oct. 1831, price 8s.

---

15. A. Cornelii Celsi de Re Medica Libri Octo, ex recensione Leon. Targæ, accedunt J. Rhodii Dissertatio de Celsi Vita, &c. Editio Secunda accuratissime emendata Opera et studio GEORGII FREDERICI COLLIER, ex Aula Magd. Oxon. E. Colleg. Med. Lond. &c. M.D. Londini, Typis Valpianis, 1831. Octavo, pp. 342.

A Translation of the eight Books of Celsus on Medicine. Second Edition, carefully revised and improved. By G. F. COLLIER, M.D. Octavo, pp. 363, 1831.

---

16. Pharmacopœia Medico-Chirurgica in Usum Medicinam facientium, a ROBERTO G. HOLLAND, Chirurgo concinnata. Edinburgi, et Londini, 1831. Octavo, pp. 185.

---

17. The History of the Contagious Cholera; with Facts explanatory of its Origin and Laws, and of a rational Method of Cure. By JAMES KENNEDY, M.R.C.S. Octavo, pp. 291. October, 1831; with a Map.

---

18. Researches to establish the Truth of the Linnæan Doctrine of Animate Contagions; wherein the Origin, Causes, Mode of Diffusion, and Cure of Epidemic Diseases, Spasmodic Cholera, Dysentery, Plague, Small-pox, Hooping-cough, Leprosy, &c. are illustrated by Facts from the Natural History of Mankind, of Animals, and of Vegetables, and from the Phenomena of the Atmosphere. By ADAM NEALE, M.D. Physician to His Majesty's Forces, &c. Octavo, pp. 258, with a Plate. October, 1831.

---

19. Caution to the Public; or Hints upon the Nature of Scarlet Fever, designed to show that this Disease arises from a peculiar and absolute Virus, and is specifically infectious in its mildest as well as in its most malignant Form; including cursory Remarks upon Plague, and other Pestilential Diseases. London, October, 1831. Octavo, pp. 165. Highley, Fleet-street.

---

20. A Memorial presented to the Medical and Surgical Officers of the Worcester, Salop, Birmingham and other Infirmaries, on the Abuses existing in the public Hospitals, &c. Octavo, pp. 12. 1831.

☞ *We fear Mr. Webb is somewhat visionary in his schemes and proposals. They will never answer.*

---

21. Elements of Anatomy, General, Special, and Comparative. From the Encyclopœdia Britannica, seventh Edition. By DAVID CRAIGIE, M.D. Quarto, double Columns, pp. 187, with 14 Plates.

☞ *This is compiled from the best authorities, with immense industry and great judgment.*

---

22. An Ordo Verborum of the First and Third Books of Celsus, &c. carefully arranged by G. F. COLLIER, M.D. Duodecimo, pp. 113. Simpkin and Marshall, 1832.

---

23. Considerations pratiques sur certaines Affections de l'Uterus, en particulier sur la Phlegmasie Chronique, avec Engorgement du Col de cet Organe, et sur les Avantages de l'Application immediate des Sangsues methodiquement employée dans cette Maladie. Par J. N. GUILBERT, M.D. Octavo, pp. 126. Paris, 1831.

---

24. A Conspectus of Prescriptions in Medicine, Surgery, and Midwifery, including the new French remedies, &c. Third Edition enlarged and improved, duodecimo, pp. 216. Simpkin and Co. October, 1831.

25. Observations on the healthy and diseased Conditions of the Breast-milk; the Disorders frequently produced in Mothers by suckling; and numerous illustrative Cases, proving that, when protracted, it is a common Cause, in Children, of Water in the Brain, &c. By Edward Morton, M.D. Octavo, pp. 56. 1831.

26. Lectures on Anatomy; interspersed with practical Remarks. Vol. III. By Bransby Cooper, F.R.S. Surgeon of Guy's Hospital, Lecturer on Anatomy, &c. Royal 8vo. pp. 300. Oct. 1831.

27. Part I.—The Principles and Practice of Obstetric Medicine, in a Series of Systematic Dissertations on Midwifery, and on the Diseases of Women and Children, illustrated by numerous Plates. By David Davis, M.D. M.R.S.L. Professor of Midwifery in the University of London, &c. Quarto, pp. 24, with a Plate. October, 1831. Price 2s. per Part.

28. An Essay on the Epidemic Cholera of India. By Reginald Orton, Surgeon, H. P. late of His Majesty's 34th Regiment of Foot. Second Edition, with a Supplement. Octavo, pp. 488, with a large Map, price 15s. Burgess and Hill, November, 1831.

☞ *See the article Cholera.*

29. Remarks on the Cholera Morbus, containing a Description of the Disease, its Symptoms, &c. By H. Young, M.D. formerly of the Hon. C. S. in Bengal. Octavo, pp. 78. Nov. 1831. Price 3s.

30. Observations on the Nature and Treatment of the Cholera Morbus, now prevailing epidemically in St. Petersburg. By George W. Lefevre, M.D. Physician to the British Embassy. Octavo, pp. 96. November, 1831.

31. The London Manual of Medical Chemistry, comprising an Interlinear Verbal Translation of the Pharmacopœia, with extensive Chemical, Botanical, Therapeutical, and Nosological Notes, &c. By William Maughan, Surgeon and Lecturer on Chemistry, &c. Duodecimo, pp. 422. November, 1831. Price 10s.

☞ *This is designed for Students, and to them it will prove extremely useful. It is multum in parvo.*

32. A practical Compendium of Midwifery; being the Course of Lectures on Midwifery, and on the Diseases of Women and Infants, delivered at Bartholomew's Hospital by the late Robert Gooch, M.D. Prepared for Publication by Geo. Skinner, M.R.C.S. Small 8vo. pp. 358, with an Index.

33. The Principles of Surgery. By J. Syme, F.R.S. &c. Octavo, pp. 347, with a Plate. Edinburgh and London, Nov. 1831.

34. Traité du Cholera Morbus, consideré sous le Rapport Medical et Administratif, ou Recherches sur les Symptomes, la Nature, et le Traitement de cette Maladie, et sur les Moyens de l'eviter. Par F. G. Boisseau, M.D. Professor at the Hospital of Instruction of Mentz, &c. Octavo, pp. 407. Bailliere, London, Nov. 1831.

☞ *This and the following are very valuable volumes, and deserve especial attention at this moment.*

Rapport sur le Cholera Morbus; lu à l'Academie Royale de Médécine, en Séance Générale, les 26 et 30 Juillet, 1831. Octavo, pp. 199. Bailliere, London.

☞ *See above.*

35. On the Propagation of Cholera. By Lieut.-Col. Rowles.

36. Traité de Chimie, appliquée aux Arts. Par M. Dumas. Tôme troisième. Octavo, pp. 784. Paris, Nov. 1831.

37. Engravings of Sir Astley Cooper, Bart. and John Abernethy, Esq. Painted by Sir Thomas Lawrence and engraved by J. Cochran. Published by Fisher and Co. London.

☞ *These are the best engravings of these two distinguished surgeons that we have yet seen.*

38. Practical Remarks on the Inutility of the Hydrostatic Test in the Detection of Infanticide. By H. W. DEWHURST, Esq. Surgeon-Accoucheur, &c. To which is added, Observations on the Employment of a new Counter-irritant in the Cure of Diseases of the Chest. Duodecimo, pp. 36, 1831. Price 1s.

---

39. A Practical Medico-Historical Account of the Western Coast of Africa:—embracing a Topographical Description of its Shores, Rivers, and Settlements, with their Seasons and comparative Healthiness; together with the Causes, Symptoms, and Treatment of the Fevers of Western Africa; and a similar Account respecting the other Diseases which prevail there. By JAMES BOYLE, M.R.C.S. Colonial Surgeon to Sierra Leone, Surg. R.N. &c. Octavo, pp. 423. Highley, London, November, 1831.

☞ *We regret that the* CHOLERA *has prevented us from reviewing this excellent Work in our present Number.*

---

40. Observations on Cholera; comprising a Description of the Epidemic Cholera of India, the Mode of Treatment and the Means of Prevention. By T. G. PETTIGREW, F.R.S. &c. Octavo, pp. 37, Highley, Nov. 1831.

---

41. The Catechism of Health; or plain and simple Rules for the Preservation of Health and the Attainment of long Life. To which are added, FACTS respecting the Nature, Treatment, and Prevention of Cholera. By A. B. GRANVILLE, M.D. F.R.S. &c. Small 8vo. pp. 336. Dec. 1831.

☞ *This little manual will do a vast deal of good among the public in general. The portion which treats of cholera will also have a very beneficial influence in diminishing choleraphobia.*

---

42. Annals of Sir Patrick Dun's Hospital. No. I. for the Year ending 5th of January, 1831. Small 8vo. pp. 72. Dublin, 1831.

☞ *To be noticed in our next.*

---

43. The History of the great Plague in London in the Year 1665; containing Observations and Memorials of the most remarkable Occurrences, both public and private, during that dreadful Period. By a CITIZEN. A new Edition, with an introductory Preface. Small 8vo. pp. 311. Renshaw and Rush, Dec. 1831.

☞ *This little volume will be read with intense interest at the present moment.*

---

44. A Treatise on Cholera Morbus, from the French of Le Baron Larrey. By HENRY PATERSON, M.R.C.S. Octavo, pp. 29, sewed. Dublin and London, Dec. 1831.

---

45. A Critical Exposition of Phrenology, being a Lecture delivered at the Philosophical Institution, Birmingham. By CHARLES CORAY, M.R.C.S. Octavo, pp. 20. Birmingham, 1831.

☞ *Mr. C. must have been somnambulant, when he sent forth this blatant supplication for notoriety.*

---

46. Lectures on the Vertebrated Animals of the British Islands:—Part I. On the British Mammalia; with a Tabular View of them, arranged according to Blumenbach's System, a Synopsis of all the Genera and Species, and an Appendix, containing a Sketch of extinct Animals. Octavo, pp. x. and 96. Birmingham and London, 1831.

☞ *This Part includes four lectures, comprising an extremely accurate natural history of the British mammiferous animals; it is composed with unusual perspicuity, conciseness, and eloquence: it abounds with evidences of intense philosophical observation and research; it contains many instructive medical and physiological suggestions: and it is animated with an excellent moral spirit, which seizes every opportunity, by reproof or sarcasm, to discourage the extravagances of those frivolous or profligate amusements, wherein a savage pleasure is elicited from the infliction of pain. We would persuade the author to complete the course of his lectures; and afterwards, having withdrawn the unopaque veil of namelessness, to re-produce them in the form of a systematic natural history.*

---

47. On Indigestion and Costiveness; with Hints to both Sexes on the important, safe, and efficacious Means of relieving Diseases of the Digestive Organs by Lavements, &c. By EDWARD JUKES, Surgeon. Octavo, pp. 196. Second Ed. Dec. 1831, with Plates.

48. Practical Examinations of the immediate Treatment of the principal Emergencies that occur in Surgery and Midwifery, systematically arranged. Intended to serve as an Exercise for the Student, and a brief Work of Reference for the General Practitioner. By W. S. OKE, M.D. Octavo, pp. 250. Longman and Co. Dec. 1831.

---

49. Elements of practical Chemistry, comprising a systematic Series of Experiments, arranged so as to form an Introduction to the Practice of Chemistry, with Directions for performing them, and for the Preparation and Application of the most important Tests and Reagents. By DAVID BOSWELL REID, M.D. &c. Second Edition, pp. 555. Maclachlan and Stewart, Edinburgh, Dec. 1831.

---

50. The Principles and Practice of Obstetric Medicine, &c. By Dr. DAVIS. Part II. price 2s. Dec. 1831.

---

51. Elements of General Pharmacy. By R. J. KANE, Professor of Chemistry to Apothecaries' Hall (Dublin), &c. Small 8vo. pp. 34, with several Plates. Dublin, Nov. 1831, price 8s. 6d.

☞ *This appears to be a very useful work.*

---

52. Medical Case-book of Record for Students and General Practitioners, &c. By W. R. R. WILTON, Surgeon, &c. for 1831.

☞ *This is the record which we use in our own private practice.*

---

53. An Essay on the Nature and Treatment of the Indian Pestilence, commonly called Cholera. By HENRY PENNECK, M.D. Octavo, sewed, pp. 40. Longman and Co. Dec. 1831.

☞ *Dr. P. considers the disease as a fever,* sui generis, *and having nothing to do with cholera.*

---

54. Memoire et Observations sur le Cholera-Morbus regnant à Varsovie. Par le Docteur F. AUTOMARCHI, Médecin de l'Empereur Napoleon à Sainte Helena, Inspecteur General des Hôpitaux Militaires en Pologne, &c. Octavo, pp. 36. Bailliere, London, 1831.

☞ *This is a very interesting pamphlet, from one who has seen the disease on a large scale.*

---

55. Atlas of Surgical Apparatus; being a Series of Delineations of the most important Mechanical Auxiliaries of Surgery, with descriptive Letter-press, explaining their several Uses and Modes of Application. By H. T. CHAPMAN, M.R.C.S. and late House-surgeon to St. Bartholomew's Hospital. Quarto, Plates, xx.; Figures, 200, lithographed. Highley, Dec. 1831.

☞ *These* 200 *figures, beautifully lithographed, represent the whole series of surgical auxiliaries in such a perspicuous manner, that the nature of the apparatus and the mode of its application are rendered evident to the meanest capacity. It is indispensable to the library of every man who undertakes the department of surgery, even on the smallest scale.*

---

56. How is cholera propagated? The Question considered, and some Facts stated. By an AMERICAN PHYSICIAN. Millar, Henrietta-street, Covent-garden. Octavo, pp. 22. Dec. 1831.

*Noticed in the Periscope.*

---

57. An Antidote to the pernicious Doctrine of Non-contagion, &c. By HENRY REID, M.R.C.S. Octavo, pp. 22. Chelmsford, Dec. 1831.

---

☞ We have not room in this number for the full titles of several works received late in the quarter—among others, Dr. Copland on Pestilential Cholera—Mr. Aldes on Iodine—Mr. Mac Notty on the Spleen—Dr. Hancock on Cholera—Dr. Norton on Diagnosis, &c. &c. &c. These will all be recorded fully in our next.

N. B. PART THE SECOND of Mr. Swan's magnificent work on the Nerves, (folio,) containing 18 plates, has been received, and will be fully noticed in our next number. It does infinite honour to the age and country in which we live.

